# The Regulation and Function of Fibroblast Growth Factor 8 and Its Function during Gonadotropin-Releasing Hormone Neuron Development

**DOI:** 10.3389/fendo.2016.00114

**Published:** 2016-09-05

**Authors:** Wilson C. J. Chung, Megan L. Linscott, Karla M. Rodriguez, Courtney E. Stewart

**Affiliations:** ^1^Department of Biological Sciences, School of Biomedical Sciences, Kent State University, Kent, OH, USA

**Keywords:** *Fgf8*, GnRH, androgen receptor, retinoic acid, epigenetics, olfactory placode

## Abstract

Over the last few years, numerous studies solidified the hypothesis that fibroblast growth factor (FGF) signaling regulates neuroendocrine progenitor cell proliferation, fate specification, and cell survival and, therefore, is critical for the regulation and maintenance of homeostasis of the body. One important example that underscores the involvement of FGF signaling during neuroendocrine cell development is gonadotropin-releasing hormone (GnRH) neuron ontogenesis. Indeed, transgenic mice with reduced olfactory placode (OP) *Fgf8* expression do not have GnRH neurons. This observation indicates the requirement of FGF8 signaling for the emergence of the GnRH neuronal system in the embryonic OP, the putative birth place of GnRH neurons. Mammalian reproductive success depends on the presence of GnRH neurons to stimulate gonadotropin secretion from the anterior pituitary, which activates gonadal steroidogenesis and gametogenesis. Together, these observations are critical for understanding the function of GnRH neurons and their control of the hypothalamus–pituitary–gonadal (HPG) axis to maintain fertility. Taken together, these studies illustrate that GnRH neuron emergence and hence HPG function is vulnerable to genomic and molecular signals that abnormally modify *Fgf8* expression in the developing mouse OP. In this short review, we focus on research that is aimed at unraveling how androgen, all-trans retinoic acid, and how epigenetic factors modify control mouse OP *Fgf8* transcription in the context of GnRH neuronal development and mammalian reproductive success.

## Introduction

Fibroblast growth factor (FGF) 8 (FGF8) signaling regulates neuroendocrine progenitor cell proliferation, fate specification, and cell survival and has therefore been hypothesized to be critical for the development and maintenance of homeostatic systems in the body such as the hypothalamus–pituitary–axis (HPG) (1–5). Indeed, FGF8 has been shown to be critical for emergence of gonadotropin-releasing hormone (GnRH) neurons (1, 2, 6). Later studies also demonstrated that FGF8 affects the postnatal maturation of vasopressin, oxytocin, kisspeptin, and corticotropin-releasing hormone neurons found in the mammalian hypothalamus (1, 2, 4, 6–8).

In humans, *Fgf8* point mutations cause Kallmann syndrome (KS), a form of congenital hypogonadotropic hypogonadism (HH) that is associated with anosmia (6, 9). Generally, KS patients do not undergo puberty and are infertile in adulthood. These observations indicate that embryonic disruptions in FGF8 signaling can abrogate GnRH neuronal development and hence cause infertility. Genetic screenings of individuals with GnRH deficiencies identified that *Fgf8* is not the only gene that is important for GnRH (progenitor) cell development, fate specification, and migration (3, 5, 10, 11). Indeed, KS patients can also harbor mutations in anosmin-1 (12–14), *fibroblast growth factor receptor-1* (*Fgfr1*) (15), *prokineticin 2* (16) and its receptor (17), and *chromodomain helicase-DNA-binding 7* (18). In this short review, we limit our discussion to the role of FGF8 signaling during GnRH neuron emergence and how *Fgf8* transcriptional activity may be controlled during olfactory placode (OP) development.

The majority of the evidence indicating that FGF8 function is required for the emergence and possibly function of GnRH neurons is derived from studies investigating how FGF8 signaling controls the emergence of the GnRH neuronal system in humans and mice (2, 3, 5, 6, 11, 19–21). FGF8 is a member of the large FGF signaling family (22) that can act through four membrane-bound tyrosine kinase FGF receptors (FGFRs) in humans and mice (23–26). Stable binding of FGFs in the presence of heparin proteoglycans enables FGFR dimerization and initiates a myriad of intracellular signal transduction pathways (27). The inherent complexity of the FGF signaling system and their functional redundancy demonstrates the robustness of FGF signaling during embryonic development (28).

**Figure 1:**
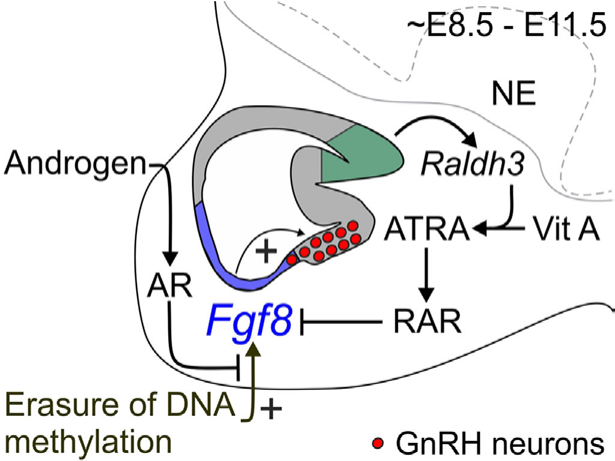
**Proposed mechanism for coordinate regulation of transient *Fgf8* transcription in the mouse OP**. Sagittal schematic representation of the ~E8.5–E11.5 mouse nasal region. FGF8 expressing cells located in the respiratory epithelium (blue region) are required for GnRH neuron emergence in the embryonic mouse mOP (red circles) located in the olfactory epithelium (gray region). Evidence from our and other studies indicates three major signaling mechanisms that regulate transient *Fgf8* expression in the embryonic mouse OP. First, our data showed that AR directly interacts with its ARE sites on the *Fgf8* promoter region. However, in contrast to our premise, DHT did not upregulate *Fgf8* expression in GT1–7 neurons or the embryonic OP. Second, inhibition of DNMT activity upregulated *Fgf8* expression suggesting that the erasure of DNA methylation induced *Fgf8* promoter activity. Surprisingly, DHT was able to block this effect, which led us to infer that androgen signaling-dependent modulation of *Fgf8* transcription in the embryonic OP is reliant on the epigenetic status of the *Fgf8* promoter. Third, ATRA signaling clearly inhibits *Fgf8* expression. The embryonic OP exhibits an age-dependent increase in ATRA levels due to the local upregulation of *Raldh3* expression [converts vitamin (Vit) A to ATRA] in the dorsal–caudal region olfactory epithelium (green region), which then acts through RARs to inhibit *Fgf8* expression. These data led us to update our hypothesis as to how *transient Fgf8* transcription is controlled in the embryonic mouse OP: upregulation of *Fgf8* transcriptional activity is DNA methylation-dependent, which is subsequently downregulated through androgen and/or ATRA signaling. In this context, androgen and/or ATRA signaling may act as a transcriptional brake to limit FGF8’s morphogenetic or proliferative effects on the embryonic mouse OP. NE, neurepithelium.

Two landmark studies published in the late 1980s have been essential in understanding where and when FGF8 signaling is critical during the development of the GnRH neuronal system (29, 30). Indeed, these studies presented convincing evidence that hypothalamic GnRH neurons in rodents and humans are born specifically in the medial OP (mOP) (Figure 1). A surprising finding, which meant that the OP morphogenetic region, which was generally known to be the anatomical precursor of the frontonasal facial structures (29, 30), also gave rise to a very specific and important hypothalamic neuroendocrine cell population.

Proliferation studies indicate that GnRH progenitor cells become postmitotic around embryonic day (E) 9.5 (30, 31). Following their emergence from the mOP, GnRH neurons migrate along the nasal septum, all the while following the olfactory, vomeronasal, and terminal nerves to the preoptic area and hypothalamus (2, 10, 30, 31). Postnatally, the majority of GnRH neurons are localized around the anterior tip of the third ventricle called organum vasculosum lamina terminalis in the preoptic area and project their axons to the external zone of the median eminence. Upon stimulation, they release the GnRH decapeptide into the portal vein system to activate gonadotropin release and production from gonadotrophs in the anterior pituitary into the systemic circulation, ultimately stimulating gonadal steroidogenesis and gametogenesis (3, 5). In the following sections, we will describe what is known about the emergence of GnRH neurons in the mOP, the role of FGF8 signaling, and how FGF8 transcription may be controlled during the mid-gestational development of the mouse OP.

## Ontogenesis of the GnRH Neuronal System

Two independent studies found that GnRH expressing neurons emerge from the mOP (30, 31). Interestingly, proliferation studies indicated that the vast majority (~80%) of mouse GnRH neurons become postmitotic in the mOP between E9.5 and E10.5 (29), which not only indicates that the birth place of GnRH progenitor cells is very likely localized in the embryonic mOP but also that GnRH-neuron fate specification occurs prior to E9.5.

The OP is an ectodermal region that gives rise to (non-sensory) respiratory epithelium and (sensory) olfactory epithelium (32–34). The olfactory epithelium develops into the main olfactory and vomeronasal systems (35, 36). Ablation studies support that the mOP is the birthplace of GnRH neurons. For example, amphibians that undergo OP removal ultimately lack the olfactory epithelium, nerve, and bulb, as well as GnRH neurons of the forebrain (37, 38). On the other hand, chick studies indicate that GnRH neurons emerge from the respiratory epithelium rather than the olfactory epithelium (39). Indeed, surgical removal of the respiratory, but not olfactory epithelium, eliminated GnRH neurons (40, 41). Mouse studies showed that the birth place of GnRH neurons may overlap with a very small portion of the respiratory epithelium that borders the olfactory epithelium (34). Overall, these data indicate that both the respiratory and olfactory epithelium contribute to the OP’s ability to generate GnRH neurons.

Migrating neural crest cells also contribute to the pool of GnRH progenitor cells. The bilateral neural crest region arises from the edge of the neural plate and shares a border with the region that eventually becomes the OP. Indeed, migrating neural crest cells contribute to the formation of the OP (42–44). As shown in zebrafish experiments, some anterior neural crest cells migrate toward the presumptive OP and contribute specific cell populations to the developing OP (45). Pre-migratory neural crest cells labeled with rhodamine dextran *in vivo* (46, 47) also double-labeled for *GnRH* mRNA and peptide (48). Similar data have also been found in mouse studies, which indicate that ~30% of the GnRH neurons found in the mOP exhibited a genetic lineage similar to neural crest cells (43, 49). The remaining 70% of the total population of GnRH neurons were confirmed to be of placodal origin. In short, the progenitor cells that develop into GnRH neurons are likely to originate from the neural crest and the OP.

## Fibroblast Growth Factors and Their Receptors

As indicated earlier, FGFs are well-known signaling proteins that are crucial for fate specification, migration, differentiation, and survival (50). There are 22 members of the FGF signaling family found in the mouse (22). Homology between FGFs is based on the presence of a highly conserved 120 amino acid core region, and the 30–60% overall amino acid identity across all FGFs (22). The presence of a N-terminal signal peptide sequence in most FGF ligands indicates that they are secreted into the extracellular milieu (51).

Fibroblast growth factor signaling is facilitated through membrane-bound tyrosine kinase receptors that contain an extracellular ligand-binding domain, a transmembrane domain, and an intracellular tyrosine kinase domain. In some instances, FGFRs translocate to or reside in the cell nucleus (23–26). Currently, four FGFRs have been identified in humans and mice. The extracellular region of a FGFR is characterized by the presence of three immunoglobulin (Ig)-like domains and one heparin-binding domain. Stable binding of FGFs in the presence of heparin sulfate proteoglycans causes dimerization of FGFRs and phosphorylation of cytoplasmic tyrosine residues in the kinase domain. This, in turn, initiates a myriad of intracellular signal transduction pathways including the phosphorylation of SH2 domain of phospholipase Cγ, hydrolysis of phosphatidylinositol bisphosphate to diacylglycerol and inositol trisphosphate, resulting in intracellular changes in Ca^2+^. FGFRs also activate the ERK pathway, specifically ERK1 and ERK2 (27). In addition, FGF signaling can activate the PI3K/AKT pathway, which in turn protects against apoptosis by inhibiting caspases (52, 53). The analysis of alternative *FGFR* mRNA splicing revealed that the third Ig-like domain comes in three versions, referred to as IIIa, IIIb, or IIIc and are expressed in a tissue-specific manner. Indeed, the IIIa form of FGFRs is secreted into extracellular environment, whereas the IIIb and IIIc forms of FGFRs are preferentially present in epithelial cells and mesenchymal cells, respectively (28). Binding affinity studies based on mitogenic activity showed that FGFRs can bind a variety of FGF ligands, indicating the presence of functional redundancy. For example, signaling of the subfamily of FGF8, 17, and 18 occurs preferentially through FGFR3c < FGFR4c < FGFR2c < FGFR1c < < FGFR3b (28). On the other hand, recent studies using surface plasmon resonance analysis detected that the binding affinity of the FGF8b splice variant (i.e., Kd) to FGFR1≈FGFR2≈FGFR3 indicating that FGF8 signaling may be mediated equally by FGFR1, FGFR2, or FGFR3 (54). The complexity and redundancy in FGF ligands and receptors demonstrates the inherent robustness of FGF signaling during embryonic development.

## FGF8 is Required for GnRH Neuron Emergence

Studies in humans and rodents have confirmed the concept that FGF8 signaling is critically important for the emergence of the GnRH neuronal system (1, 2, 6, 20, 21). Indeed, GnRH neurons are absent in homozygous *Fgf8* hypomorphic embryonic and newborn mice (2, 6). Not surprisingly, the number of GnRH neurons was also reduced in heterozygous *Fgf8* hypomorphs when compared to wildtype mice (2, 6). Later studies further showed that the deficit in GnRH neurons delayed puberty in female mice, even though recent studies indicate that FGF8 dysfunction did not reduce adult male or female hypothalamic GnRH peptide synthesis, except on postnatal day 60 (4, 21). Together, these results provided a fundamental explanation for the reproductive defects found in KS patients who harbor hemizygous *Fgf8* mutations (55).

The elimination of GnRH neurons likely occurred during the emergence phase of GnRH neuronal development (~E9.5–E10.5). Currently, we do not know whether the elimination of the GnRH progenitor cells is due to abrogated FGF8-dependent proliferation or cell survival. However, evidence favors the second possibility given that the presence of increased apoptosis has been reported in the E10.5 OP of mice with reduced *Fgf8* expression (5). Moreover, studies in conditional *Fgf8* null mice found that apoptosis in the OP was much higher in wildtype mice on E10.5. In contrast, no difference was found in the level of cell proliferation (56). Therefore, attenuated FGF8 expression in the early embryonic OP likely abrogated the survival of GnRH (progenitor) cells.

Studies in the chicken embryos confirmed that the timing of FGF8 signaling is critical for the emergence of the GnRH in the OP (57), which occurs around Hamburger and Hamilton (HH: time after egg-laying) 19. HH17 chick OP explant cultures will express GnRH mRNA after ~2 days in culture, which the FGFR antagonist SU5402 prevented. On the other hand, exogenous FGF8 induced and could advance chick GnRH mRNA expression (57). These studies confirmed our conclusion that the timing of transient FGF8 signaling is critical for the emergence of GnRH neurons in the OP (2). Interestingly, exogenous FGF8 had unexpected effects on mouse GT1–7 GnRH-secreting neurons. These studies showed that treatment with the most abundant FGF8b splice form inhibited GnRH mRNA expression (58). These contrasting data sets could indicate that FGF8 affects GnRH neuron development in a bi-phasic fashion. First, FGF8 initially acts as a neurotrophic factor to prevent the elimination of GnRH progenitor cells, thereby allowing the emergence of the GnRH neuronal system from the mOP. Second, it has to be recognized that FGF8 also functions as morphogenetic factor that maintains the proliferative character of progenitor cells (59–63). Therefore, continued FGF8 function on GnRH neurons could result in the dedifferentiation of fate-specified cells, such as GT1–7 neurons.

Recent cell lineage studies confirmed that *Fgf8* mRNA is primarily localized in the respiratory epithelium (64). More importantly, these studies also indicate that GnRH neurons are not derived from *Fgf8* expressing respiratory epithelium progenitor cells (11, 32). Therefore, we conclude that *Fgf8* expressing respiratory epithelial cells may provide trophic support, which promotes the survival of E9.5–E10.5 GnRH progenitor cells in the mOP (Figure 1).

## Androgen Effects on Transient OP *Fgf8* Transcription

Earlier studies indicate that mouse OP *Fgf8* mRNA expression upregulates around embryonic day (E) 8.5 followed by a downregulation around E11.5/12.5 (61, 63, 65, 66). FGF8 was originally discovered as an androgen-induced growth factor in SC-3 cells, an androgen-dependent mouse breast cancer cell line (67). Studies also showed that testosterone induced *Fgf8* mRNA in LNCaP cells (68). *In vitro* analysis showed that androgen-bound androgen receptors (ARs) act on androgen response elements (AREs) to activate *Fgf8* promoter-coupled luciferase activity, which was inhibited with the anti-androgens (69). Functional deletion studies showed that androgen activates *Fgf8* transcription through these putative AREs in the *Fgf8* promoter (69). Our data confirmed that the embryonic mouse OP expresses high AR mRNA levels, while ChIP qPCR quantification found that AR interacts with three AREs on the mouse *Fgf8* promoter in GT1-7 neurons and OP explants, in an androgen-independent fashion (70). These results led us to conclude that unliganded AR may be responsible for the upregulation of OP *Fgf8* transcription between E8.5 and E11.5, to stimulate GnRH (progenitor) cell emergence/survival. However, our recent studies in mouse OP explants showed that androgen did not modulate *Fgf8* nor did it affect *GnRH* mRNA expression (70) suggesting that androgen, unlike in SC-3 or LNCaP cells, is not the primarily regulator of *Fgf8* transcription in the embryonic mouse OP. However, as discussed later, we cannot fully rule out the possibility that androgen signaling effects on *Fgf8* expression depends on the epigenetic status of the *Fgf8* promoter (Figure 1).

## Retinoic Acid Effects on Transient OP *Fgf8* Transcription

On the other hand, it is clear that all-trans retinoic acid (ATRA) signaling is a strong inhibitor of *Fgf8* expression in the vertebrate OP (Figure 1). For instance, ATRA-soaked beads proximal to the chick OP suppresses virtually all nearby *Fgf8* expression, which was correlated with a coincident elimination of emerging GnRH neurons (57). The inhibition of OP *Fgf8* expression is thought to be caused by local synthesis of ATRA by RA-synthesizing aldehyde dehydrogenase 3 (RALDH3) from vitamin A in OP cells (Figure 1), which in turn activates retinoid receptors, such as RA receptor (RAR) α, β, γ in the developing OP (11, 57, 71). RARs bind ATRA and the stereoisomer 9-cis RA, whereas RXRs only bind to 9-cis RA (72). Although RARs and RXRs act as heterodimeric complexes that affect gene transcription, studies indicate that RXRs primarily act as silent partners with respect to gene transcription (72–74). Moreover, 9-cis RA is absent in the embryonic mouse (72). Data from developmental brain studies clearly showed that ATRA acts to spatially and temporally restrict *Fgf8* mRNA expression to achieve polarity in body axis (75, 76). Recently, our preliminary data showed that ATRA prevents (unpublished results) the normal upregulation of GnRH expression found in mouse OP explants (70). Therefore, we conclude that ATRA acting through RARs temporally restricts *Fgf8* transcription in the mouse OP region, consequently causing the downregulation of OP cell *Fgf8* mRNA levels after E11.5/E12.5.

## Epigenetic Effects on Transient OP *Fgf8* Transcription

As previously mentioned, our recent studies showed that the regulation of *Fgf8* transcription is under the control of DNA methylation, a conclusion that is supported by our recently published results (70). Multiple CpG islands upstream and downstream of the translation start site of the *Fgf8* gene have been detected (http://www.ncbi.nlm.nih.gov/geo). The presence of these CpG islands in the *Fgf8* gene suggests that *Fgf8* transcriptional and mRNA expression in OP cells may be DNA methylation-dependent. DNA methylation depends on DNMTs, which catalyze cytosine methylation in the presence of the methyl group donor *S*-adenosyl methionine (71). Evidence from previous studies indicates that E11.5 olfactory progenitor cells express high levels of DNMTs (77). Specifically, mitotic olfactory progenitors express exclusively DNMT3b, whereas postmitotic olfactory neurons express DNMT3a (77). Interestingly, semiquantitative analysis showed that *Dnmt3b* mRNA expression is gradually reduced after E11.5 (77). Together with the rise of *Fgf8* transcription between E8.5 and E11.5, we inferred that the *Fgf8* gene may initially be heavily methylated followed by a process of DNA demethylation, which is in line with studies indicating that DNMT repression in E7.5–E10.5 mice causes global DNA demethylation, subsequently upregulating global gene expression (78, 79). Therefore, we predicted that reducing DNMT activity would induce *Fgf8* expression. To investigate this possibility, we treated GT1-7 neurons with AZA for 3 days and found that the AZA increased *Fgf8* mRNA levels (70). These data support that erasure of DNA methylation due to reduced DNMT-mediated maintenance may induce *Fgf8* transcriptional activity (80). Currently, we are exploring whether active DNA demethylation facilitated by ten-eleven translocation enzymes potentiates embryonic erasure of DNA methylation in the mouse OP, which has not been studied before.

We do not know whether AZA directly controls DNA methylation status of the *Fgf8* promoter; however, previous studies indicating that hypomethylation in primary rhabdomyosarcoma tumors was correlated with higher *Fgfr1* mRNA expression levels (81). Our current research focuses on verifying whether DNA demethylation of the *Fgf8* promoter regions is the underlying molecular mechanism that causes the upregulation of OP *Fgf8* expression, possibly through the inhibition of DNMT activity. On the other hand, we cannot rule out the possibility that AZA indirectly elicited the upregulation of *Fgf8* expression, such as inducing or inhibiting the expression of AR or RARs.

To our surprise, the non-aromatizable androgen, DHT, eliminated the AZA-dependent rise in *Fgf8* mRNA levels, which is the basis of our earlier inference that androgen signaling depends on the epigenetic status of the *Fgf8* promoter region. Based on these results, androgen signaling modulation of *Fgf8* expression may act in concert with other changes in cellular milieu during OP development. One possibility is that, in the context of epigenetic modification of *Fgf8* expression, androgen signaling through the AR may act as a transcriptional brake that limits the morphogenetic or proliferative effects of FGF8 signaling due to DNA demethylation-dependent upregulation of *Fgf8* expression. For instance, a possibility is that the demethylation of the *Fgf8* promoter allows the constitutively-bound ARs to recruit co-repressors in the presence of DHT. However, this is highly speculative as there are no data supporting this idea. Nonetheless, these results indicate that increased DNA demethylation can induce *Fgf8* transcription and that androgen signaling is functionally repressive.

## Summary and Conclusion

Our recent research indicates that transient OP *Fgf8* transcription may be due to the coordinated actions of AR, RARs, and DNA demethylation, which together provide a permissive transcriptional milieu that initially promotes and later limits the emergence of GnRH neurons. Indeed, disruption of transient *Fgf8* transcription in the OP can eliminate the emergence of the entire GnRH neuronal system suggesting that FGF8’s primary function is to provide trophic support for the emerging GnRH neurons. However, data from GT1-7 neurons indicate that FGF8 causes a reduction in GnRH mRNA expression. Taken together, we conclude that the physiological function of transient *Fgf8* transcription is twofold. Initially, the upregulation of FGF8 promotes and provides trophic support required for the emergence of GnRH neurons in the mOP. However, because FGF8 is also known to maintain the undifferentiated nature of progenitor cells, FGF8 expression in the OP region must be downregulated before it dedifferentiates the newly emerged GnRH neurons. Based on the presented data, we speculate that the coordinated control of transient *Fgf8* transcription acts as a molecular system that spatially and temporally controls not just the emergence of GnRH neurons (i.e., total number) but also whether they remain GnRH neurons. In essence, we conclude that transient FGF8 is possibly a major molecular cue that controls the number GnRH neurons that can emerge from the mOP, which could affect the functionality of the GnRH neuronal system as it pertains to the HPG-axis and reproductive success.

## Author Contributions

All authors contributed to the conceptual development and writing of this review.

## Conflict of Interest Statement

The authors declare that the research was conducted in the absence of any commercial or financial relationships that could be construed as a potential conflict of interest.
